# Influence of planting density on growth performance, salt dynamics, and nitrogen uptake in *Suaeda salsa* L

**DOI:** 10.3389/fpls.2026.1770729

**Published:** 2026-03-27

**Authors:** Xinzhi Feng, Yanyan Wang, Jinxiu Hao, Ke Zhang, Wenxuan Mai, Wentai Zhang, Ahmad Azeem

**Affiliations:** 1 College of Resources and Environment, Xinjiang Agricultural University, Urumqi, China; 2 Xinjiang Institute of Ecology and Geography, Chinese Academy of Sciences, Urumqi, China; 3 College of Resources and Environment, Yili Normal University, Yining, China

**Keywords:** biomass, halophyte, planting density, saline alkali soil, salt removal

## Abstract

**Introduction:**

Optimal planting density (PD) is a critical agronomic practice that directly influences plant growth and yield. *Suaeda salsa L.* can absorb salts from saline soils and accumulate them in its tissues, thereby playing a significant role in saline-alkali soil remediation. However, studies on the effects of *S. salsa* PD on soil improvement are limited.

**Methods:**

In this study, a pot experiment was conducted with three planting densities, two plants per pot (D1), four plants per pot (D2), and six plants per pot (D3), to determine their effects on the growth and salt accumulation in *S. salsa*. The soil moisture in each pot was maintained at 80% of the field capacity and was continuously controlled throughout the growing period using a weighing method.

**Results:**

The results showed that PD significantly influenced the morphological traits and salt removal capacity. Throughout the growing season, plant height, leaf area, and biomass were highest under D2, exceeding those under D1 by 29–78% and those under D3 by 1–6%. Nitrogen accumulation first increased and then decreased with increasing PD, reaching its maximum under D2 treatment. Although the shoot Na^+^ concentration decreased over time, the overall biomass continued to increase, resulting in a gradual increase in total salt accumulation, which peaked at the fruiting stage. Under D2, the highest salt removal was observed, reaching nearly 4.17 g pot^-1^ (84.44 g m^-2^).

**Discussion:**

The optimal density for *S. salsa* was 4 plants per pot (81 plants m^-2^), which led to greater salt removal and higher biomass accumulation.

## 1 Introduction

China possesses 3.63 × 10^7^ hm^2^ of saline-alkali soil, accounting for 4.88% of its available land area (Hopmans et al., 2021). Xinjiang accounts for the largest share, with 1.34 × 10^7^ hm^2^, representing 36.80% of the total area (Zhang et al., 2025). As a significant strategic reserve of arable land, rational and sustainable utilization of saline-alkali soils is vital for sustaining long-term agricultural productivity. Therefore, the scientific and systematic management of these soils is essential to ensure stable socioeconomic development and national food security (Liu and Wang, 2021; Cheng et al., 2023). Currently, China advocates an integrated approach that aligns crop selection with land conditions while adapting land use to meet the specific requirements of the crops. This strategy effectively integrates the principles of “storing grain in land” and “storing grain through technology.” This framework facilitates comprehensive remediation and efficient utilization of saline-alkali land. Among the available strategies, the cultivation of halophytes or salt-tolerant crops is widely regarded as one of the most effective approaches for the productive utilization of saline soils (Jabborova et al., 2023).

*Suaeda salsa* L., an annual euhalophyte, is widely distributed in intertidal zones along China’s eastern coastline and across inland arid and semi-arid regions (Wang and Song, 2019). This species maintains normal growth under saline-alkali conditions and can accumulate large quantities of salt, making it particularly suitable for the biological remediation of saline-alkali soils (Hayat et al., 2020). In arid areas, harvesting the aboveground biomass of *S. salsa* can remove up to 5.185 kg hm^-2^ of salt from the soil during a single growing season (Zhao et al., 2013). A three-year field experiment conducted by (Wang L. et al. (2021). demonstrated that the mean annual salt removal capacity reached 3.839 kg hm^-2^, a level sufficient to compensate for salt inputs associated with irrigation. In coastal areas, the cultivation of *S. salsa* has been shown to significantly decrease the soluble salt content in moderately vegetated plots compared with that in unvegetated tidal flats (Chen et al., 2021). Furthermore, its potential as a forage crop provides additional economic value (Wang et al., 2023; Abebe and Tu, 2024). However, existing studies on the salt-removal capacity of *S. salsa* have not fully considered the influence of planting density (PD) on salt accumulation efficiency.

PD plays a pivotal role in regulating crop growth and development, with non-optimal densities potentially causing yield losses of up to 65% (Shao et al., 2021). Stress induced by inappropriate PD often results in morphological changes, such as stunted growth, reduced yield, and modifications in physiological and biochemical processes, including inhibited metabolic activity, reduced photosynthetic efficiency, and lowered respiratory rates (Huang et al., 2021). Therefore, determining the optimal PD is essential for achieving stable and high crop yields in agricultural production systems (Testa et al., 2016). In natural habitats, the plant architecture of *S. salsa* is strongly influenced by PD. When density reaches or exceeds 1,088 plants m^-2^, plant height (PH) is limited to approximately 40 cm with minimal branching; in contrast, at densities below 10 plants m^-2^, PH can increase up to 120 cm (SHAO et al., 2004). In alfalfa, PD is closely associated with the forage yield. Appropriate density maximizes individual plant yield potential, whereas excessively low or high densities lead to reduced overall yield performance (Liu et al., 2024). Under drip irrigation conditions, PD has been shown to exert a significant effect on the yield of *S. salsa* (Pan et al., 2014). As a typical halophyte, *S. salsa* exhibits distinct adaptive responses to varying salinity levels. Wang et al., (2024a) reported that the cultivation of *S. salsa* significantly reduced soil salinity within the 0–20 cm soil layer. Specifically, in soils with light to moderate salinity-alkali levels (0.6–1.1%), aboveground sodium removal accounted for 12.1–19.3% of the total soil sodium content. Conversely, when salinity increased to 2.6%, this proportion declined sharply to 4.3%. Notably, maximum sodium removal was observed at a salinity of 1.1%. Furthermore, it has been reported that under excessively low salinity conditions, the specialized salt regulation metabolism mechanisms of *S. salsa* may not be fully activated, resulting in impaired growth or even reduced survival due to competitive disadvantages. Based on these findings, we selected a 1% salinity level for this study to represent a moderate-to-high saline stress environment. This level not only effectively stimulates salt accumulation and adaptive physiological mechanisms in *S. salsa* but also corresponds to its strong growth adaptability and ecological restoration potential for saline-alkali land remediation. Collectively, these findings suggest that PD regulation can optimize plant morphology and enhance biomass production and salt accumulation in *S. salsa*. Despite numerous studies documenting the influence of PD on halophyte biomass and ion accumulation, there is a notable lack of synchronous quantitative evidence detailing the complex relationship linking “PD, stand growth, soil salinity dynamics, nitrogen (N) uptake, and partitioning” under controlled saline-alkali conditions. Prior studies have largely focused on individual parameters or lacked continuous monitoring of soil salinity fluctuations throughout the entire growth cycle, thereby limiting the mechanistic understanding of how PD modulates salt translocation processes and their subsequent impact on the acquisition of N. Therefore, this study aimed to systematically evaluate the effects of different planting densities on the growth performance and salt removal capacity of *S. salsa*, providing a scientific foundation for its efficient application in saline-alkali land restoration. This study hypothesizes that PD influences the upward movement of salts and rhizosphere salinity by altering stand structure, thereby regulating N uptake and allocation. Simultaneously, the transfer of effects between the per-plant and per-unit-area scales may create trade-offs among biomass, salt removal, and N uptake, resulting in an optimal density range.

## 2 Materials and methods

### 2.1 Experimental materials

**Figure 1:**
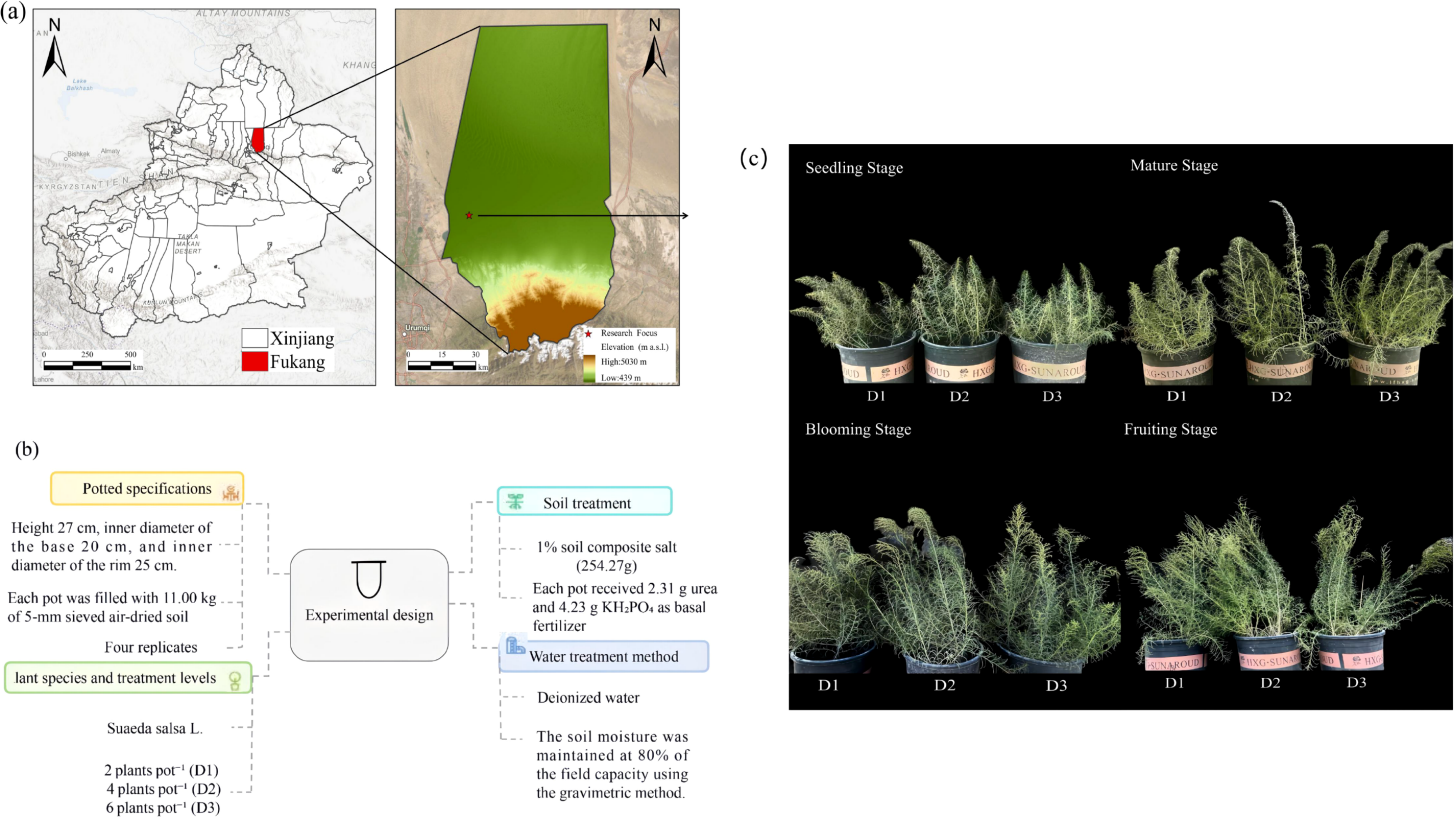
**(a)** Study area; **(b)** Experimental Design. **(c)** Different growth stages.

Seeds of *S. salsa* were collected from the Halophyte Botanical Garden (84°59′41.61″ E, 45°28′6.38″ N) in Karamay, Xinjiang, China. Soil samples were obtained from a long-term abandoned cotton field at the Fukang Desert Ecological Station, Chinese Academy of Sciences (87°45′–88°05′ E, 43°45′–44°30′ N). The initial physical and chemical properties of the soil are presented in **Table 1**. The experiment was conducted using pots measuring 27 cm in height, with inner bottom and top diameters of 20 and 25 cm, respectively (**Figure 1**).

**Table 1:** Initial soil physical and chemical properties.

Soil depth (cm)	Available N (mg kg^&−1^)	Available phosphorus (mg kg^&−1^)	Available potassium (mg kg^&−1^)	pH	Total dissolved salt (g kg^&−1^)	Electronic conductivity (mS cm^&−1^)
0-20cm	59.02	34.13	664.10	7.82	1.65	0.39

### 2.2 Experimental design

This experiment was conducted at the Fukang Desert Ecological Station, Chinese Academy of Sciences (44.28° N, 87.93° E), from May 26 to September 29, 2024, under a 4 m high rain shelter with a light transmission rate exceeding 80%. Each pot received 2.31 g of urea (CO(NH_2_)_2_) and 4.23 g of KH_2_PO_4_ as basal fertilizers to support the growth of *S. salsa*. Subsequently, 254.27 g of a compound salt (composition shown in **Table 2**, collected from the edge of the Gurbantunggut Desert) was dissolved in 2.0 L of deionized water and applied to each pot to adjust the soil salinity to 1.0%. The soil moisture was adjusted to approximately 80% of the water-holding capacity of the containers. The experiment consisted of three PD treatments: D1 (two plants per pot), D2 (four plants per pot), and D3 (six plants per pot), with 16 replicates for each. After 24 hours, 20 intact seeds of *S. salsa* were uniformly sown in each pot and covered with a 2 mm layer of air-dried soil. The seedlings were thinned to the target densities when they reached a height of approximately 6 cm. Throughout the growing period, the soil moisture was maintained at 60% of the water-holding capacity of the container using gravimetric weight measurements.

**Table 2:** Salt content and ion composition of salt crust.

Salt crust	pH	EC	TDS	CO_3_^2−^	HCO_3_^−^	Cl^−^	SO_4_^2−^	Ca^2+^	Mg^2+^	Na^+^	K^+^
(cm)		(mS cm^−1^)	(g kg^−1^)	(g kg^−1^)							
0-5	9.65	70.46	432.62	0.51	0.24	11.73	261.18	2.99	4.64	114.27	0.20

Salt crust, Salt crust thickness; EC, Electrical conductivity; TDS, Total dissolved solids; CO_3_^2−^, Carbonate; HCO_3_^−^, Bicarbonate; Cl^−^, Chloride; SO_4_^2−^, Sulfate; Ca^2+^, Calcium; Mg^2+^, Magnesium; Na^+^, Sodium; K^+^, Potassium.

### 2.3 Sampling and measurement

The growth period of *S. salsa* was divided into four stages: seedling (60 days), mature (80 days), blooming (90 days), and fruiting (119 days). Sampling was conducted once at each stage, with four replicate pots for each treatment. Before sampling, the PH, stem diameter (SD), and leaf area (LA) of *S. salsa* were measured at each growth stage using a measuring tape. After measuring the growth traits, plants from each replicate of each treatment were harvested for further analysis. After harvest, the leaves, stems, and roots of each plant in every replicate were separated and immediately oven-dried at 105 °C for 30 minutes, then dried at 75 °C for 48 hours until a constant weight was achieved. Total biomass was calculated as the sum of the dry weights of the roots, stems, and leaves.

Soil samples were air-dried, ground, and sequentially sieved through 2 mm and 1 mm meshes before analysis. Soil pH and electrical conductivity (EC) were measured in deionized water extracts at soil-to-water ratios of 1:2.5 (w/v) for pH and 1:5 (w/v) for EC using a pH meter (S20, Mettler-Toledo, Switzerland) and a conductivity meter (DDSJ-308A, China). The total dissolved solids (TDS) were determined using the residue-drying method. Plant samples (roots, stems, and leaves) were ground in a ball mill, digested with HNO_3_-H_2_O_2_ in a microwave digestion system, and analyzed for sodium (Na) content using inductively coupled plasma atomic emission spectrometry (ICP-AES; 735E, Agilent, USA).

### 2.4 Statistical analysis

The normality of the data was assessed using the Shapiro-Wilk test. A one-way analysis of variance was conducted to evaluate the differences in soil TDS content, biomass, PH, SD, LA, Na^+^ concentration, and Na^+^ removal. The least significant difference test was used for *post-hoc* multiple comparisons. All statistical analyses were performed using Microsoft Excel and SPSS version 29.0 (IBM Corp., Armonk, NY, USA). Correlation analysis was conducted using OriginPro 2022 for the growth trial data. Graphs were generated using OriginPro 2022.

## 3 Results

### 3.1 Effects of PD on *S. salsa* height

**Figure 2:**
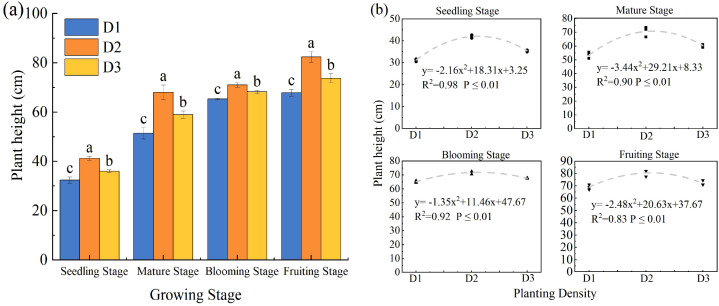
Effect of planting density on plant height of *S. salsa***(a)** and curve fitting **(b)**. Note Vertical error bars represent ± standard error (N = 4). Different lowercase letters indicate statistically significant differences between different treatments at the same sampling time (p < 0.05). 2 plants pot^−1^ (D1), 4 plants pot^−1^(D2), and 6 plants pot^−1^(D3).

Throughout the entire growth period of *S. salsa*, PD significantly influenced PH (*p* < 0.05; **Figure 2a**), with values first increasing and then decreasing as the density increased. D2 density produced the greatest height, exceeding D1 by 8.60–32.36% and D3 by 2.30–17.20%. PH exhibited a clear quadratic response to PD (**Figure 2b**), indicating that four plants per pot was the optimal density for achieving maximum height.

### 3.2 Effects of PD on stem diameter of *S. salsa*

**Figure 3:**
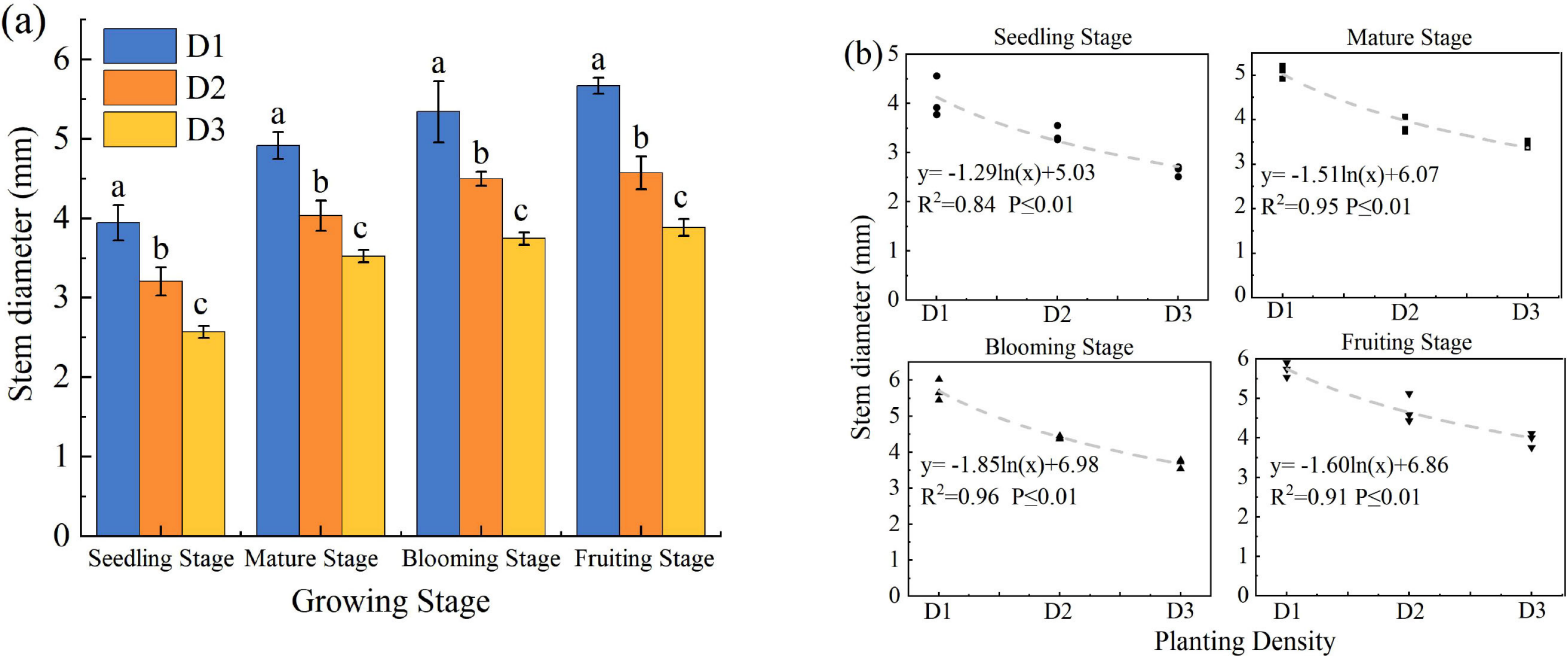
Effect of planting density on stem diameter of *S. salsa***(a)** and curve fitting **(b)**. Note vertical error bars represent ± standard error (N = 4). Different lowercase letters indicate statistically significant differences between different treatments at the same sampling time (p < 0.05).2 plants pot^−1^(D1), 4 plants pot^−1^(D2), and 6 plants pot^−1^ (D3).

SD decreased significantly with increasing PD throughout the growth period (*p* < 0.05; **Figure 3a**). Low density significantly enhanced stem thickening (*p* < 0.05), whereas high density significantly inhibited it. The SD in D1 was 1.19–1.24 times and 1.39–1.53 times greater than that in D2 and D3, respectively. A logarithmic model best described the relationship between SD and PD, indicating a non-linear, negative correlation (**Figure 3b**).

### 3.3 Effects of PD on LA of *S. salsa*

**Figure 4:**
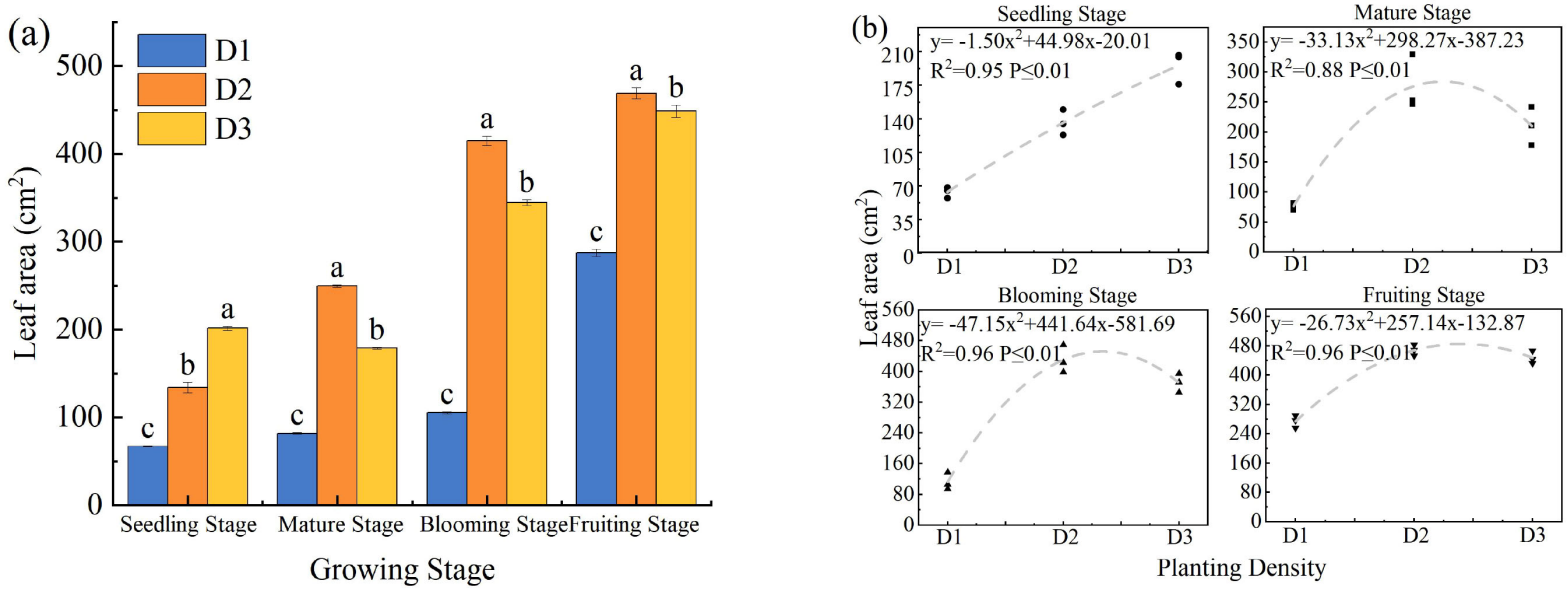
Effect of planting density on leaf area of *S. salsa***(a)** and curve fitting **(b)**. Note vertical error bars represent ± standard error (N = 4). Different lowercase letters indicate statistically significant differences between different treatments at the same sampling time (p < 0.05). 2 plants pot^-1^ (D1), 4 plants pot^-1^ (D2), and 6 plants pot^-1^ (D3).

PD significantly affected the LA of *S. salsa* at all growth stages (*p* < 0.05; **Figure 4a**). The LA exhibited an overall trend of first increasing and then decreasing with increasing density. During the seedling stage, LA gradually increased as PD increased. In the later growth stages, that is, maturity, blooming, and fruiting, the LA peaked at the intermediate PD (D2), showing significantly higher values compared with the D1 and D3 treatments by 70.49% to 280.99% and 63.06% to 228.05%, respectively (*p* < 0.05; **Figure 4a**). Quadratic regression analysis revealed a significant quadratic relationship between LA and PD (**Figure 4b**).

### 3.4 Effects of PD on biomass and its allocation in *S. salsa*

**Figure 5:**
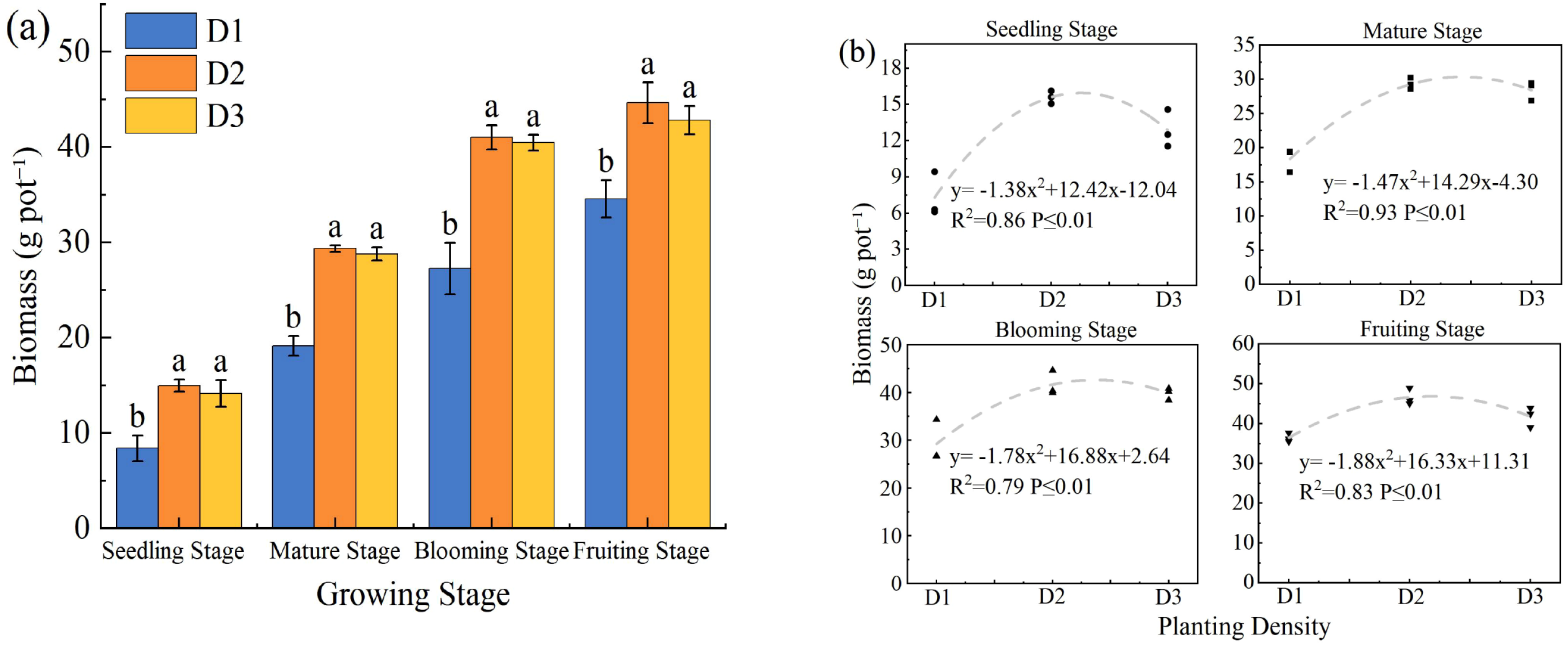
Effect of planting density on biomass of *S. salsa***(a)** and curve fitting **(b)**. Note vertical error bars represent ± standard error (N = 4). Different lowercase letters indicate statistically significant differences between different treatments at the same sampling time (p < 0.05). 2 plants pot^−1^ (D1), 4 plants pot^−1^ (D2), and 6 plants pot^−1^ (D3).

PD had a significant quadratic effect on *S. salsa* biomass (*p* < 0.05). Biomass initially increased and then decreased at higher density (**Figure 5a**). Density level D2 consistently produced the highest biomass, which was 29–78% greater than that of D1 and 1–6% greater than that of D3. Regression analysis revealed a quadratic relationship between biomass and PD (**Figure 5b**), indicating that maximum biomass occurred at 4–5 plants per pot.

**Figure 6:**
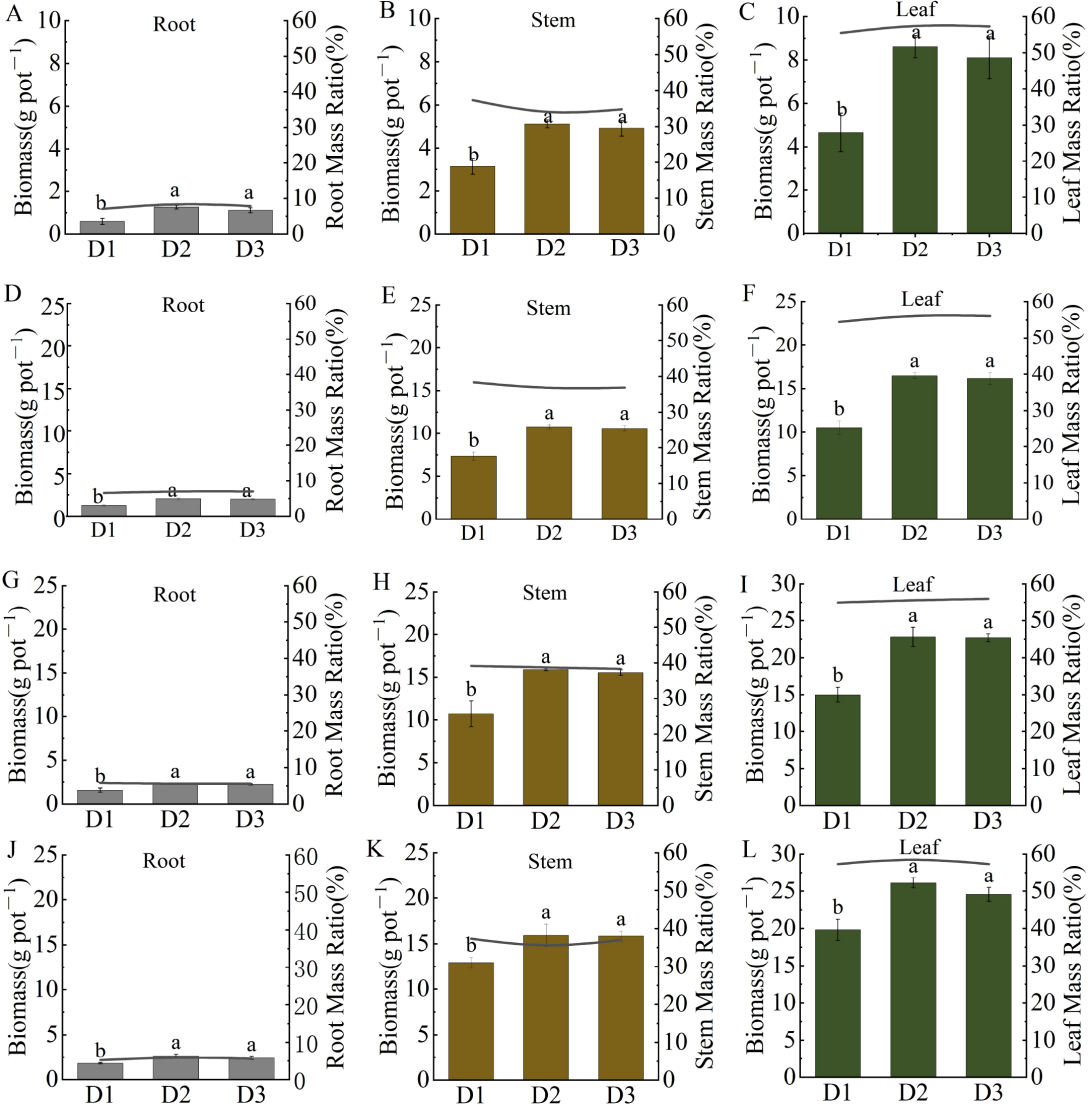
Effects of planting density on biomass allocation in different organs Biomass allocation and mass ratios of roots **(A, D, G, J)**, stems **(B, E, H, K)**, and leaves **(C, F, I, L)** at different sampling stages.Note Vertical error bars represent ± standard error (N = 4). Different lowercase letters indicate statistically significant differences between different treatments at the same sampling time (p < 0.05).

PD significantly influenced biomass allocation among the roots, stems, and leaves of *S. salsa*, following a consistent pattern of leaves > stems > roots (**Figure 6**). At the seedling stage, compared with D1, D2 increased root, stem, and leaf biomass by 110%, 63%, and 84%, respectively. The D3 treatment increased these biomasses by 87%, 56%, and 73%, respectively. At the fruiting stage, biomass increased under the D2 treatment by 44% (roots), 23% (stems), and 32% (leaves), whereas under the D3 treatment, increases were approximately 32%, 23%, and 24%, respectively. Among all the organs, leaf biomass exhibited the greatest increase. Although the D2 and D3 treatments showed similar growth performance, D2 demonstrated slightly superior overall biomass accumulation.

### 3.5 Effects of PD on TDS and salt removal of *S. salsa*

**Figure 7:**
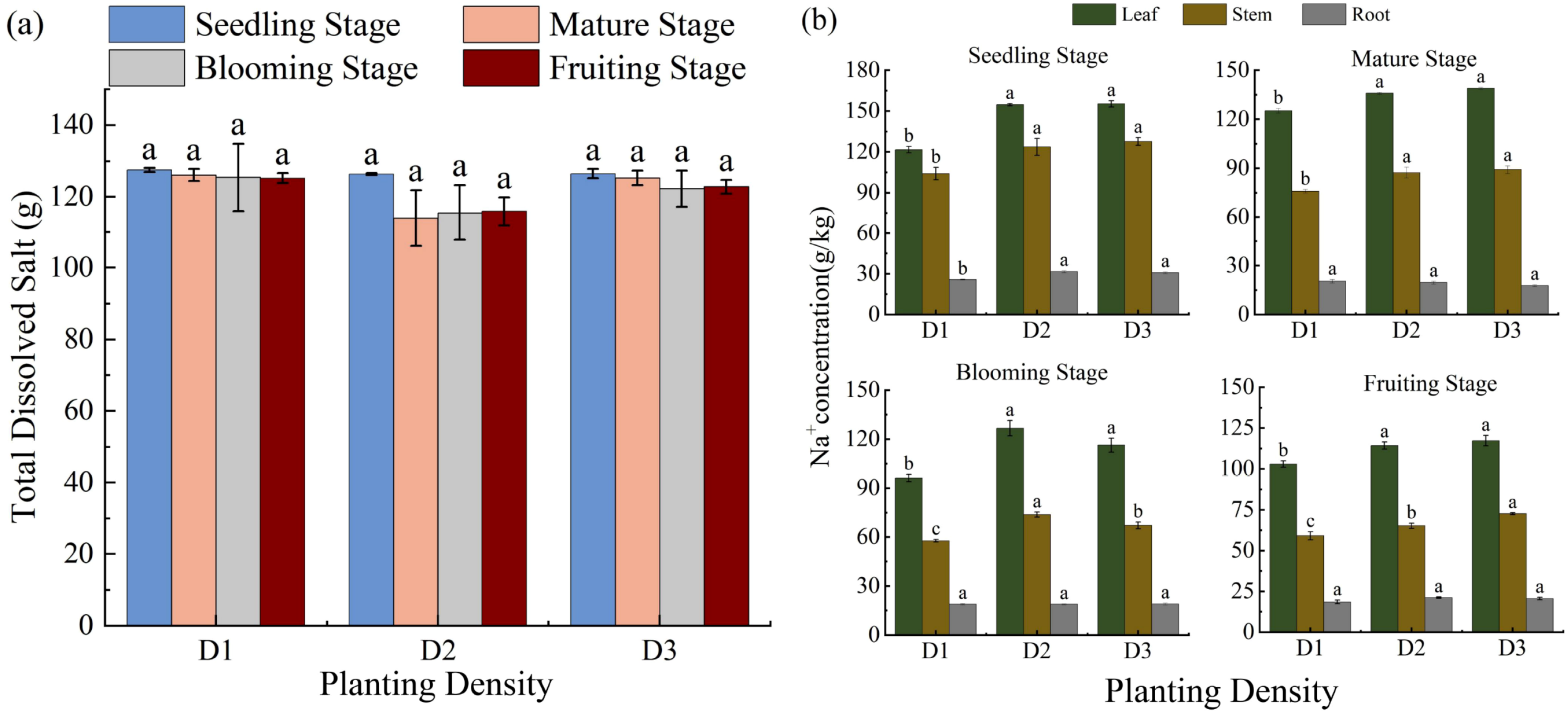
Effects of planting density on total dissolved salts **(a)** and Na^+^ concentration in S. salsa soil **(b)**. Note: vertical error bars represent ± standard error (N = 4). Different lowercase letters indicate statistically significant differences between different treatments at the same sampling time (p < 0.05). 2 plants pot^−1^(D1), 4 plants pot^−1^(D2), and 6 plants pot^−1^(D3).

TDS exhibited a nonlinear response to PD over time, characterized by an initial decrease followed by an increase. At the seedling stage, D2 treatment most effectively reduced soil TDS from 128.15 g to 113.99 g. In contrast, D3 treatment reduced the soil TDS to 122.25 g, representing a 4.60% decrease (**Figure 7a**). These results indicate that the reduction in soil TDS is closely related to the salt removal capacity of *S. salsa*.

The Na^+^ concentration in the vegetative tissues of *S. salsa* increased significantly with increasing PD (*p* < 0.05), following the order: leaf > stem > root. The leaves were the primary organs for salt accumulation, followed by the stems (**Figure 7b**). Biological salt removal was calculated by multiplying the Na^+^ concentration in the stems and leaves by their respective biomasses. Maximum desalination occurred at the fruiting stage, followed by the blooming and seedling stages. Notably, at a PD of four plants per pot, Na^+^ removal reached 4.17 g per pot, accounting for approximately 3.6% of the added salt content. Although *S. salsa* exhibited measurable salt-removal capacity, its efficiency in the pot experiment remained relatively limited.

### 3.6 Effect of PD on N concentration and accumulation in salt-affected *S. salsa*

**Figure 8:**
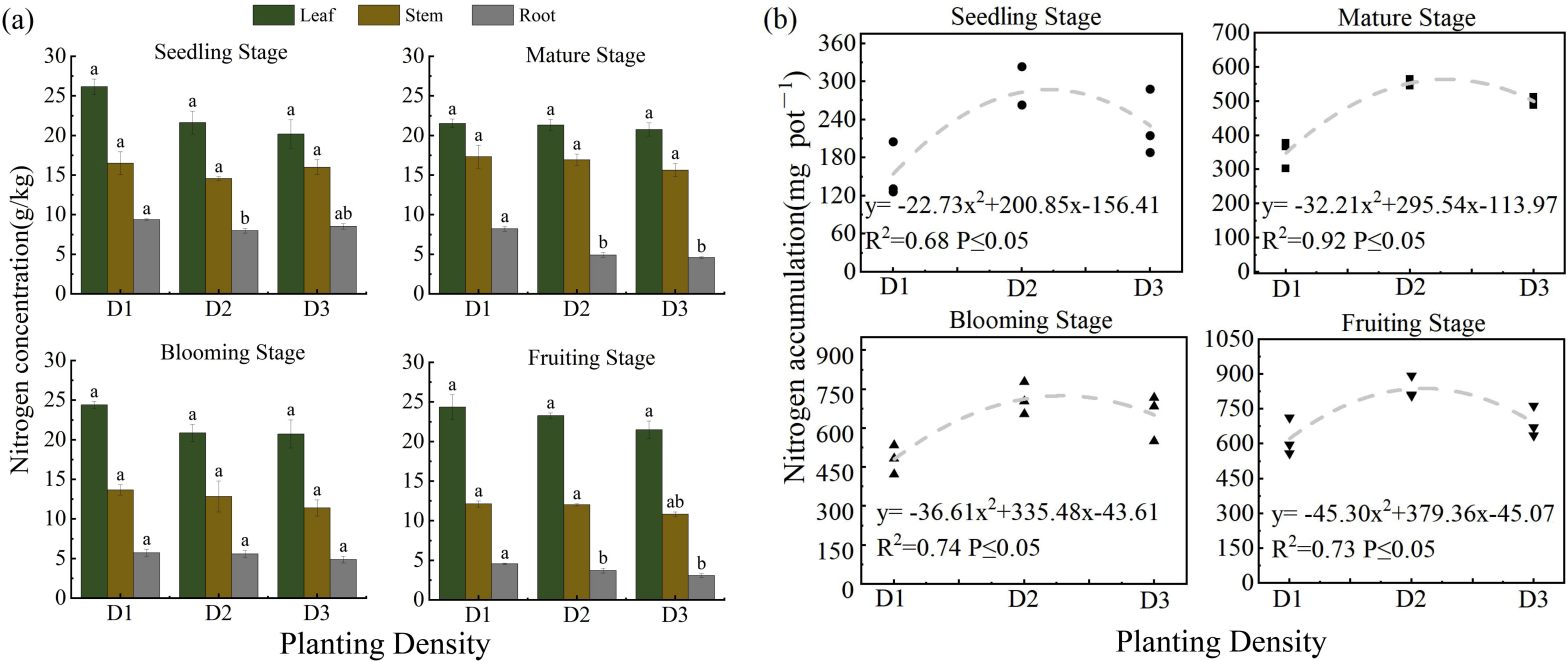
Effect of planting density on nitrogen concentration **(a)** and nitrogen accumulation **(b)**. Vertical error bars represent ± standard error (N = 4). Different lowercase letters indicate statistically significant differences between different treatments at the same sampling time (p < 0.05). 2 plants pot^−1^(D1), 4 plants pot^−1^ (D2), and 6 plants pot^−1^(D3).

Throughout the entire growth period of *S. salsa* in saline-alkaline soil, different planting densities significantly affected the N content and accumulation in the plants (*p* < 0.05). As PD increased, the N content and accumulation first increased and then decreased. Among the treatments, D2 resulted in the highest N concentration (**Figure 8a**) and total N accumulation in all *S. salsa* organs. In addition, N accumulation exhibited a clear quadratic response to varying planting densities (**Figure 8b**), further indicating that a PD of four plants per pot was optimal for maintaining N levels.

### 3.7 Correlation between PD and morphological traits of *S. salsa*

**Figure 9:**
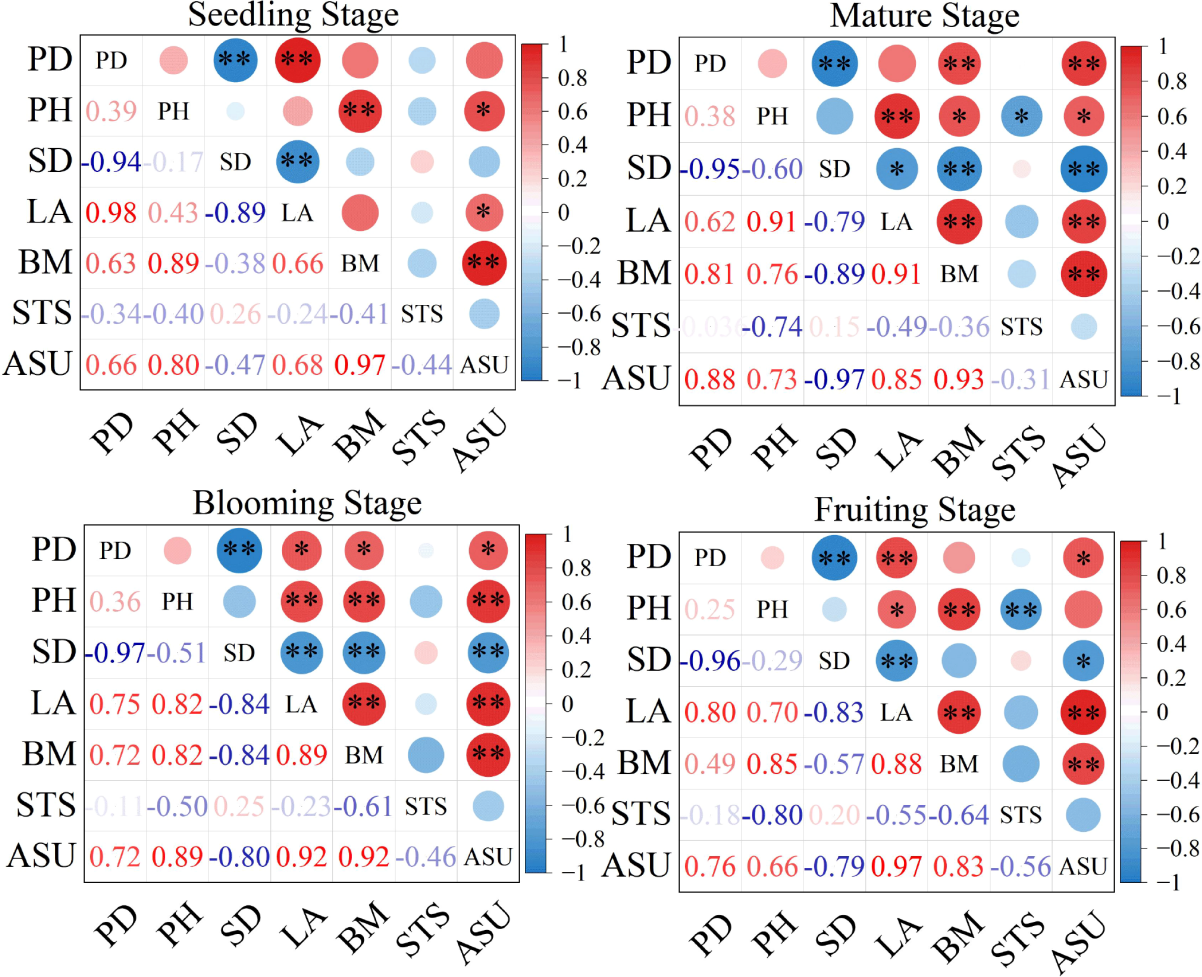
Correlation analysis of *S. salsa* with different planting densities and different growth stages **(a)** Seedling stage, **(b)** Mature stage, **(c)** Blooming stage, **(d)** Fruiting stage. PD, planting density; PH, plant height; SD, stem diameter; LA, leaf area; BM, biomass; TDS, total dissolved salt; ASU, aboveground salt uptake; NA, Nitrogen accumulation. 2 plants pot^−1^ (D1), 4 plants pot^−1^(D2), and 6 plants pot^−1^(D3). *p<0.05, *p<=0.01.

Correlation analysis of the morphological traits of *S. salsa* across growth stages revealed that SD was significantly and negatively correlated with PD at all stages (*p* ≤ 0.01; **Figure 9**). A larger SD was significantly associated with reduced LA, biomass (BM), and salt uptake. LA showed a significant positive correlation with BM (*p* ≤ 0.01) throughout all growth stages, particularly during the blooming and fruiting stages. Similarly, PH exhibited a significant positive correlation with aboveground biomass and salt uptake (*p* ≤ 0.01), particularly at the seedling stage, where greater PH promoted increased LA values. Therefore, PD played a crucial regulatory role in shaping plant morphology. Under high-density conditions, although stem thickening was inhibited, PH, LA, and aboveground salt uptake increased significantly, peaking during the blooming stage of the plant.

### 3.8 Effects of PD on root-to-shoot ratio and specific LA

**Figure 10:**
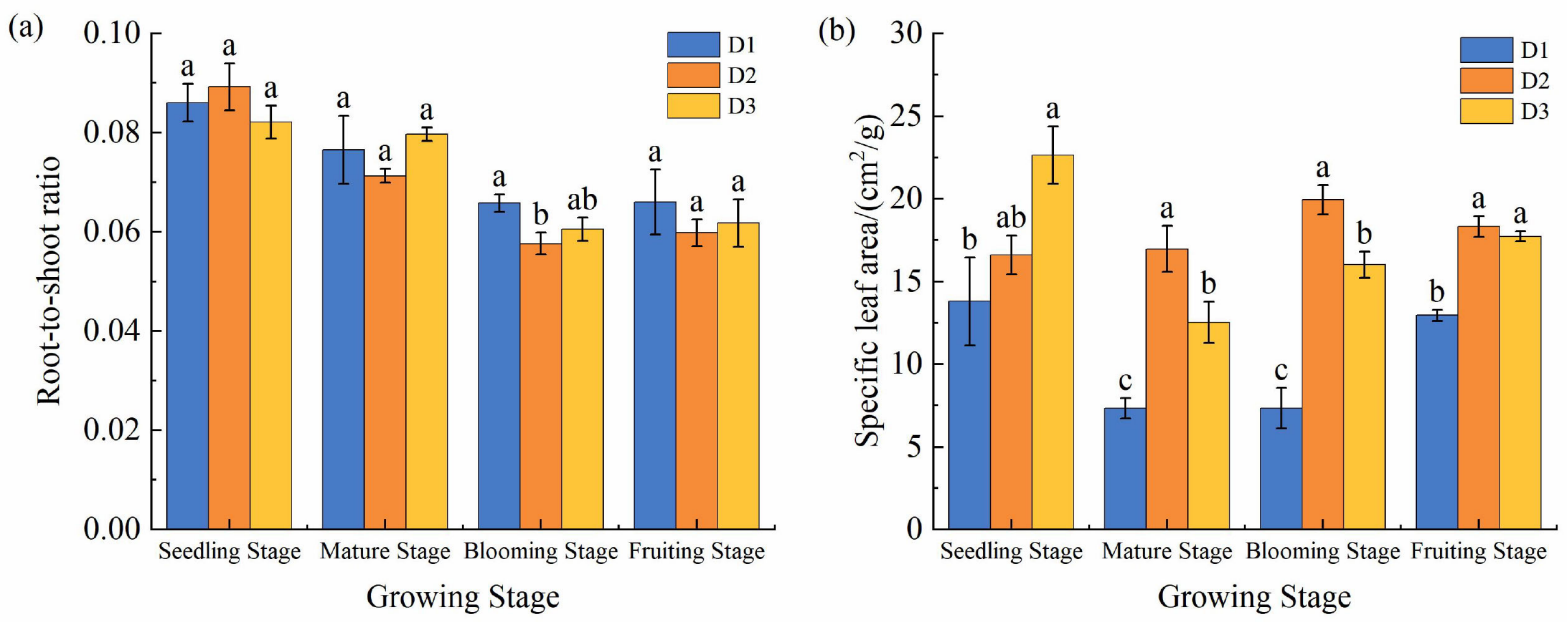
Effects of planting density on the root-to-crown ratio **(a)** and the specific leaf area **(b)**. Vertical error bars represent ± standard error (N = 4). Different lowercase letters indicate statistically significant differences among treatments at the same sampling time (p < 0.05). 2 plants pot^−1^ (D1), 4 plants pot^−1^(D2), and 6 plants pot^−1^(D3).

The results indicate that PD significantly affected the root-to-crown ratio and specific LA of *S. salsa* (*p* ≤ 0.05; **Figure 10**). Except during the seedling stage, the root-to-crown ratio under the D2 treatment was significantly lower than that under the other treatments at all other growth stages, whereas SLA generally increased with PD before decreasing. For example, during the fruiting stage, under D2, the root-to-crown ratio of *S. salsa* decreased by 9.4% compared to that under D1 and by 3.24% compared to that under D3, whereas its SLA increased by 41.41% relative to that under D1 and by 3.37% relative to that under D3.

## 4 Discussion

PD is a crucial environmental factor that influences plant community growth and resource use efficiency. Biomass, as a key indicator of production capacity and resource accumulation in plant communities, effectively reflects the growth potential of plants under specific density conditions (Kang et al., 2020). In this study, the biomass of *S. salsa* exhibited a typical quadratic response to increasing PD, with the D2 treatment achieving the highest biomass, which was significantly greater than that of D1, whereas D3 showed a slight decline (**Figure 5**). This pattern, an initial increase followed by a decrease with increasing density, is consistent with previous findings (Zhang et al., 2021). In the D1 treatment, weak intraspecific competition facilitated favorable individual growth (**Figures 2**–**4**), but the low plant density limited the total biomass accumulation (**Figure 6**). As PD increases, community productivity improves despite reduced individual growth (Chai et al., 2024), indicating that the yield can be enhanced through higher plant numbers. However, under D3 treatment, intensified competition reduces canopy light transmittance and photosynthetic efficiency, ultimately diminishing the growth advantage of the community (Niu et al., 2014). Therefore, moderate intraspecific competition promotes efficient resource utilization, minimizes resource waste, enhances the coordinated development of root and canopy systems, and establishes a beneficial balance between competition and cooperation, thereby maximizing community biomass (Wang et al., 2011).

Furthermore, an increase in PD exacerbates resource competition, prompting plants to adapt through morphological adjustments (Wang et al., 2017). In this study, agronomic traits such as PH, LA, and SD were key indicators of plant growth performance, resource use efficiency, and ecological adaptability. Their responses to PD exhibited fundamentally different curve types, which revealed the unique growth strategies and resource allocation trade-offs of the plants (Xu et al., 2023). Specifically, PH and LA initially increased and then decreased at higher densities, presenting as a quadratic unimodal curve, that is, a “single-peak” response (**Figures 2**, **4**) (Lupa-Condo et al., 2024). From D1 to D2, PH and LA increased significantly, likely due to moderate-density facilitative effects within groups or morphological adjustments to maximize light acquisition. During this phase, plants invest more in vertical growth and leaf expansion to optimize resource capture. However, in the D3 treatment, overcrowding led to a decline in growth, reflecting intensified intraspecific competition that suppressed growth. For example, under D1, plants exhibit lateral expansion and branching, which limit vertical growth and reduce the efficiency of nutrient allocation. In contrast, in the D3 treatment, intense competition induced resource scarcity, prompting *S. salsa* to rapidly elongate its stems to obtain more light. This response eventually results in mutual shading, reduced photosynthetic efficiency, and constrained root development, producing tall and fragile plant structures (Wang LL. et al., 2021).

Additionally, SD decreased with increasing density, following a logarithmic pattern characterized by a “decreasing nonlinear negative correlation” (**Figure 3**). This trend aligns with the site-density rule (Wu et al., 1996), further elucidating the plant resource allocation strategy in resource-limited and highly competitive environments. The primary functions of stems include mechanical support, water and nutrient transport, and storage. To gain an advantage in light competition, plants strategically allocate limited resources, often at the expense of stem thickening. The continued reduction in SD indicates its relatively low priority in resource allocation, whereas the decelerating rate of decline suggests that it is approaching the minimum biological limit necessary to maintain the basic structure and function. This pattern clearly reflects the trade-off between PD and structural robustness.

Aboveground biomass accumulation is a key determinant of the salt removal capacity of *S. salsa* and is significantly and positively correlated with salt-uptake efficiency. Under identical salinity conditions, plant communities with higher aboveground biomass and elevated salt ion concentrations generally exhibit greater overall salt absorption capacity. Although *S. salsa* accumulates relatively high Na^+^ concentrations under saline stress, its total salt removal remains limited because of growth inhibition induced by salinity (Wang et al., 2024a). Similarly, *Salicornia europaea* exhibits higher tissue salt concentrations than *S. salsa*, but its reduced aboveground biomass results in substantially lower salt accumulation per unit area (Wang et al., 2022). These findings indicate that species with greater biomass production can achieve higher net ion sequestration, demonstrating enhanced salt-removal potential. Leaves serve as the primary sites for salt storage in *S. salsa*, accounting for more than 50% of the aboveground biomass, and the Na^+^ concentrations in leaves significantly exceed those in other organs. This characteristic aligns with the regionalized salt storage mechanism (Liao et al., 2007), whereby plants mitigate ion toxicity by sequestering salts in leaf vacuoles.

PD modulates root absorption capacity and N metabolic processes, thereby shaping nutrient distribution patterns in *S. salsa* grown in saline-alkali soils. In this study, we observed that N accumulation exhibited a quadratic response to PD under salt-stress conditions. Because N accumulation depends on both biomass and N concentration, this quadratic pattern likely results from the combined effects of the “population scale effect” and the “resource limitation effect.” Specifically, within the D1–D2 density range, an increase in plant number led to higher collective biomass and total N uptake, resulting in an upward trend in N accumulation. However, under D3 treatment, limited pot space and intensified inter-plant competition for N, water, rhizosphere space, and light resources became prominent. Furthermore, salt stress exacerbates this situation by affecting ammonification and nitrification in the soil; specifically, Cl^-^ competes with NO_3_^-^, and NH_4_^+^ competes with Na^+^, creating ion toxicities and imbalances that directly limit N uptake, transport, and assimilation. These combined effects amplify competition-induced N limitation under high-density conditions, resulting in reduced individual plant growth and N uptake. Consequently, the increase in collective N accumulation slowed or even declined, following a quadratic pattern. This observation is consistent with the findings of Kumar et al (Kumar et al., 2024), who highlighted that increased PD significantly affects soil N availability and uptake owing to heightened inter-plant nutrient competition, thereby supporting the density-driven N dynamics observed in this study under salt stress conditions.

Studies have shown that N distribution in *S. salsa* follows the pattern leaf > stem > root, indicating that N is primarily concentrated in photosynthetic organs to support metabolic activities. In this study, the N concentration in plants was highest under the D1 treatment and slightly lower under D3 than D2; however, owing to the reduced biomass in the D1 treatment, N accumulation was highest under the D2 treatment. Wang et al., (2024b). reported that an appropriate PD promotes N absorption and utilization by crops, whereas excessively high PD reduces soil oxygen, inhibits root growth and N uptake, and ultimately lowers yield and N use efficiency. Furthermore, as PD increases, the N concentration in various organs decreases, suggesting that at D3 PD, nutrient distribution coordination within the plant is weakened (Liu et al., 2025). In addition, the effect of PD on salt absorption capacity further supports these conclusions. Density significantly affected the salt absorption capacity of *S. salsa*, primarily by modulating aboveground biomass and per-plant salt uptake efficiency. Salt absorption at the population level exhibited a quadratic response, initially increasing and then decreasing with increasing PD (Xu et al., 2024). From D1 to D2, the increased density enhanced population biomass, particularly under D2, where the aboveground biomass was significantly higher than that under D1 (**Figure 6**), thereby strengthening the salt storage capacity and resulting in maximum salt removal. A similar pattern has been reported, for example, where N fertilization increases ash content by promoting biomass accumulation (Wang  and Tian, 2011). However, under D3 treatment, excessively high density suppressed plant growth and development, thereby constraining photosynthetic assimilation and organic matter accumulation (Begna et al., 1999), which is consistent with the observed reduction in biomass. Additionally, although the Na^+^ concentration was highest under D1 treatment, the limited number of plants resulted in lower overall desalination. The Na^+^ concentration progressively decreased as the PDs increased (**Figure 7**). Consequently, under the D2 treatment, despite not showing the highest Na^+^ concentration, the synergistic effect of substantial biomass and more efficient resource utilization enabled the highest salt removal of 4.17 g per pot.

However, single-cycle salt removal accounted for only approximately 3.6% of the added salt. This limited proportion resulted from the enclosed pot design of the experiment, where salt export depended almost entirely on plant uptake and removal through harvested biomass. This finding reflects the single-cycle removal efficiency of *S. salsa* via biological pathways without physical leaching, highlighting its unique ion-removal capacity. From a long-term perspective, repeated planting and standardized harvesting hold promise for increasing cumulative salt export. However, their sustainability is constrained by multiple factors, including seasonal biomass production, ion accumulation capacity, external salt input, and resalinization. Consequently, a more pragmatic application of *S. salsa* involves its integration as a component of a “salt absorption and export” strategy, achieving cumulative net removal through multi-seasonal continuous cultivation and seasonal biomass harvesting. In field practice, halophytes should be synergistically combined with water and salt management practices, such as drainage and leaching. This integrated approach establishes a stable and efficient salt export pathway: continuous ion absorption by plants reduces soil solution salinity, which, in turn, maintains or enhances leaching efficiency and effectively mitigates resalinization.

This study, conducted in pots under controlled saline-alkali conditions, provides valuable insights into the differential responses of *S. salsa* growth, salt removal, and N uptake at different planting densities. However, certain inherent limitations must be acknowledged. First, restricted soil volume and confined root growth space may alter plant strategies for water, salt, and N acquisition, thereby affecting the intensity of intraspecific competition. Second, although the soil salinity gradient under pot conditions is more uniform and controllable, complex natural processes, such as evaporation, precipitation/leaching, groundwater recharge, and field spatial heterogeneity, are difficult to fully simulate. This limitation directly affects the extrapolation of soil salinity dynamics and phytodesalination efficiency under field conditions. Therefore, the “optimal density” derived from this study should be regarded as an equivalent reference value obtained under controlled conditions, and its applicability in the field requires further validation through trials conducted under diverse salinity/alkalinity gradients and irrigation/drainage management regimes.

## 5 Conclusions

Regulating PD effectively optimizes the architectural traits of *S. salsa*, thereby significantly influencing its biomass accumulation and salt removal capacity. As PD increased, both biomass and salt uptake exhibited a unimodal pattern, initially increasing and then decreasing, with peak values achieved at a density of four plants per pot. Based on these findings, it is recommended to revise the current high-density planting practices under field conditions by adopting a moderate-density planting strategy. Specifically, population density should be maintained at four plants per pot^-^¹ in controlled experiments, or approximately 81 plants m^−2^ under field conditions (this value is derived by normalizing to the pot-opening area and is intended solely for unit-based comparison rather than as a direct field recommendation) to optimize individual plant growth and maximize salt removal efficiency.

## Data Availability

The raw data supporting the conclusions of this article will be made available by the authors, without undue reservation.
